# Advanced Driving Assistance Based on the Fusion of Infrared and Visible Images

**DOI:** 10.3390/e23020239

**Published:** 2021-02-19

**Authors:** Yansong Gu, Xinya Wang, Can Zhang, Baiyang Li

**Affiliations:** 1 School of Information Management, Wuhan University, Wuhan 430072, China; guyansong@whu.edu.cn (Y.G.); lby_whu@whu.edu.cn (B.L.); 2 Electronic Information School, Wuhan University, Wuhan 430072, China; zhangcan@whu.edu.cn

**Keywords:** advanced driving assistance, infrared and visible image fusion, smart city, generative adversarial network

## Abstract

Obtaining key and rich visual information under sophisticated road conditions is one of the key requirements for advanced driving assistance. In this paper, a newfangled end-to-end model is proposed for advanced driving assistance based on the fusion of infrared and visible images, termed as FusionADA. In our model, we are committed to extracting and fusing the optimal texture details and salient thermal targets from the source images. To achieve this goal, our model constitutes an adversarial framework between the generator and the discriminator. Specifically, the generator aims to generate a fused image with basic intensity information together with the optimal texture details from source images, while the discriminator aims to force the fused image to restore the salient thermal targets from the source infrared image. In addition, our FusionADA is a fully end-to-end model, solving the issues of manually designing complicated activity level measurements and fusion rules existing in traditional methods. Qualitative and quantitative experiments on publicly available datasets RoadScene and TNO demonstrate the superiority of our FusionADA over the state-of-the-art approaches.

## 1. Introduction

Smart cities have become new hot spots for global city development, including smart transportation, smart security, smart communities, and so on. Among them, advanced driving assistance is an indispensable and effective tool playing a pivotal role in smart transportation. The core of a smart city is a high degree of information fusion, so as advanced driving assistance. In the advanced driving assistance scene, there are a large number of information sensing devices to monitor, connect and interact with objects and pedestrians in the environment online [1]. Among the sensors, infrared and visible sensors are generally the most widely used types of sensors whose wavelengths are 300–530 nm and 8–14 μm, respectively.

The peculiarity of combining infrared and visible sensors depends on the fact that visible image captures reflected light to represent abundant texture details, while infrared image captures thermal radiation, which can emphasize thermal infrared targets though in poor lighting conditions or under the severe occlusion [2,3,4]. Based on the strong complementarity between infrared and visible sensors, the fused results can show abundant texture details with salient thermal targets. Therefore, infrared and visible image fusion is undoubtedly a significant and effective application in advanced driving assistance, which is much more beneficial for automatic detection of the system or driver’s visual perception.

In the infrared and visible images fusion, many methods have been proposed in the past few years, and they can be divided into six categories according to corresponding schemes, including pyramid methods [5,6], neural network-based methods [7], wavelet transformation based methods [8], sparse representation methods [9,10], salient feature methods [11,12], and other methods [13]. There are three main parts in these fusion methods, i.e., (i) domain transform, (ii) activity level measurement, and (iii) fusion rule design. The biggest criticism lies in that designing complex activity level measurements and fusion rules manually are usually needed in most existing methods, which leads to additional time consumption and complexity.

The development of the smart city is inseparable from the empowerment of artificial intelligence (AI). Among them, the powerful feature extraction capabilities of deep learning have caught more and more eyes [14,15]. Some detailed exposition about these fusion methods will be discussed later in Section 2.2. These deep learning-based methods have found a new breakthrough for image fusion and also achieved excellent effects. However, this kind of method does not completely break away from the shackles of traditional methods, because the framework based on deep learning is typically only applied to some small parts, e.g., the extraction of features, while the whole fusion process is still based on traditional frameworks.

In addition, both traditional and deep learning-based methods suffer from a common predicament, i.e., information attenuation. Specifically, the extracted (or to be fused) information, including texture details and salient thermal targets, are attenuated to varying degrees due to the weight selection accompanying the fusion process.

To address the above issues and improve the performance of advanced driving assistance, in this paper, we propose a new fusion method that is fully based on deep learning, called FusionADA. For convenience, we abbreviate source visible and infrared images, and fused image as VI, IR and $I_{F}$, respectively. First of all, in our fusion model, deep learning runs through the whole model, and manually designing complex activity level measurements and fusion rules are not required, thus our FusionADA is a fully end-to-end model. Furthermore, our FusionADA can overcome the predicament of information attenuation, which is reflected in texture details and salient thermal targets, respectively. On the one hand, since the texture details can be characterized by gradient variation, based on the major intensity information, we employ the max-gradient loss to guide the fused image to learn the optimal texture details from source images. On the other hand, with a labeled mask reflecting the domains of salient thermal targets, we establish a specific adversarial framework of two kinds of neural networks, i.e., the generator and the discriminator, based on conditional generative adversarial networks (GAN). Rather than a whole image, the real data only refers to the salient thermal targets from the source infrared image limited by the labeled mask (*M*), i.e., IR⊗M, while the fake date refers to the corresponding regions of the fused image, i.e., $I_{F}⊗M$, which forces the fused image to restore the salient thermal targets from the source infrared image. In conclusion, our FusionADA can be trained to generate the fused image with the optimal texture details and salient thermal targets in a fully end-to-end way without information attenuation.

The main contributions of this paper can be summarized into two aspects as follows. (i) In order to improve the performance of the advanced driving assistance, we propose a new fully end-to-end infrared and visible images fusion method, which is achieved without any manual designs of complex activity level measurements and fusion rules. (ii) To overcome the predicament of information attenuation, we employ the max-gradient loss and adversarial learning to learn the optimal texture details and restore the salient thermal targets, respectively.

The rest of this paper is arranged as follows: In Section 2, we present some related works with a conspectus of explanations of the advanced driving assistance and existing deep learning-based fusion methods. The detailed introduction of our FusionADA with the motivation is presented in Section 3. Section 4 shows the fusion performance of our FusionADA on public infrared and visible image fusion datasets RoadScene and TNO, compared with other state-of-the-art methods in terms of both qualitative visual effect and quantitative metrics. Besides, we carry out the ablation experiment of adversarial learning in this section, followed by some conclusions in Section 5.

## 2. Related Work

In this section, we provide brief explanations of the advanced driving assistance in smart transportation and deep learning-based fusion methods.

### 2.1. Advanced Driving Assistance

Advanced driving assistance refers to a kind of integrated system that integrates a camera detection module, a communication module and a control module, which is of great benefit for vehicle driving tasks. Specifically, there are different operating principles and levels of assistance to the drivers. The advanced driving assistance can be divided into different classes according to the monitored environment, and the used sensors [16]. These systems will not act completely autonomously, they will only provide relevant information to drivers and assist them when taking key actions. The proposed infrared and visible images fusion method relies on exteroceptive sensors, and the information of fused results is shown on a screen as the visual assistance to the drivers, which can be also incorporated in automatic recognition by smart transportation.

### 2.2. Infrared and Visible Image Fusion Based on Deep Learning

In the last several years, the breakthroughs in deep learning have driven the vigorous development of artificial intelligence, which also provides new ideas for infrared and visible image fusion. Fusion methods based on deep learning can be roughly divided into two categories: convolutional neural networks (CNN)-based model and GAN-based model [17]. In the methods based on CNN, Liu et al. [18] firstly established a deep convolutional neural network to achieve the generations of both activity level measurement and fusion rule, which are also applied for fusing infrared and visible images. Innovatively, Li et al. [19] used the architecture of dense block to get more useful features from source images in the encoding process, followed by a decoder to reconstruct the fused image. Besides, a novel convolution sparse representation was introduced by Liu et al. [20] for image fusion, where a hierarchy of layers was built by deconvolutional networks. As for the methods based on GAN, Ma et al. [21,22] proposed the FusionGAN to fuse infrared and visible images by adversarial learning, which is also the first time that the GANs are adopted for addressing the image fusion task. Xu et al. [23] achieved fusion via a conditional generative adversarial network with dual discriminators (DDcGAN), in which a generator accompanied by two discriminators is employed to enhance the functional information in IR and texture details in VI.

## 3. Proposed Method

In this section, combining the characteristics of infrared and visible images and the fusion target, we give a detailed introduction to the proposed method, including our fusion formulation, the network architectures of generator and discriminator, and the definitions and formulations of loss functions.

### 3.1. Fusion Formulation

**Figure 1:**
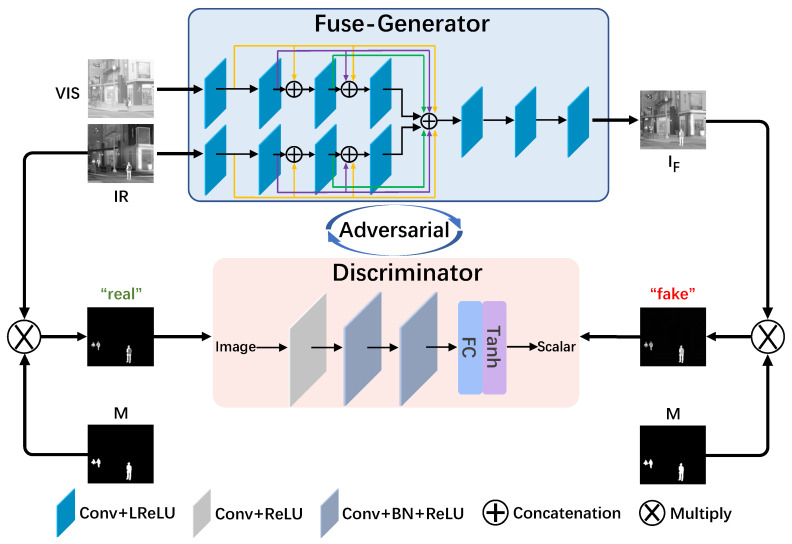
The training procedure of FusionADA.

The training procedure of our proposed FusionADA is illustrated in Figure 1. The infrared images can distinguish the targets from their background based on the dissimilarity in thermal radiation, but they lack rich texture details. In contrast, the visible images are able to show relatively richer texture details with high spatial resolution, but they fail to highlight the salient targets. Besides, for a certain area corresponding to the two source images, the infrared or visible image may own better texture details. Given an infrared image IR and a visible image VI, the ultimate goal of our FusionADA is to learn a fuse-generator *G* conditioned on them constrained by a content loss. With the labeled mask *M* reflecting the domain of salient thermal targets, the fused image $I_{F}$ multiplied by the labeled mask *M*, i.e., $I_{F}⊗M$ is encouraged to be realistic enough and close enough to real data, i.e., $I_{R}⊗M$, to fool the discriminator *D*. Meanwhile, the discriminator aims to distinguish the fake data ($I_{F}⊗M$) from the real data ($I_{R}⊗M$). Accordingly, the objective function of adversarial learning can be formulated as follows:

(1) $$\min_{G}\max_{D}E\left[\log D\left(I_{R}⊗M\right)\right]+E\left[\log\left(1-D\left(I_{F}⊗M\right)\right)\right].$$

After the continuous optimization of the generator and the adversarial learning of the generator and the discriminator, the fused image will finally possess the optimal texture details and salient thermal targets in a fully end-to-end way.

### 3.2. Network Architecture

**Fuse-Generator *G*:** As shown in Figure 1, the Fuse-Generator can be regarded as an En-decoder structure. In the encoder, for each image, we use a branch to extract information from it. Adopting the idea of DenseNet [19], each layer is directly connected with other layers in a feed-forward manner. Since the information extracted from each source image is not the same, the internal parameters of each branch are also different. There are four convolutional layers in each branch, and each convolutional layer consists of the operations of padding and convolution, and the corresponding activation function, i.e., leaky rectified linear unit (LReLU). In order to avoid the blurring of the image edges caused by “SAME”, the padding mode of all convolution layers is set as “VALID”. The additional padding operation placed before convolution is employed to keep the size of feature maps unchanged and match the size of source images. The kernel sizes of the first two convolutional layers are set to 5, while the kernel sizes of the latter two convolutional layers are set to 3. The strides of all convolutional layers are set to 1. Since the number of output feature maps of each convolutional layer is 16, the number of the final concatenated output feature maps is 128.

The decoder is used for channel reduction and fusion of the extracted information. The kernel sizes in all convolutional layers are uniformly set to 1 with the strides setting to 1, and thus the sizes of feature maps will not change. Therefore, there are no padding operations. The activation function of the last convolutional layer is set as Tanh. Moreover, the specific settings for the number of output channels in all layers are summarized in Table 1.

**Discriminator *D*:** The discriminator is added to form an adversarial relationship with the generator. The input of the discriminator is the real data, i.e., $I_{R}⊗M$, or the fake data, $I_{F}⊗M$, and the output is the scalar estimating the probability of the discriminator’s input image from real data rather than fake data. There are only three convolutional layers in the discriminator, which is much simpler compared to the Fuse-Generator. The strides of all convolutional layers are set to 2. After the fully connected layer, the scaler is obtained by the activation function Tanh.

### 3.3. Loss Functions

The loss functions in our work are composed of the loss of Fuse-Generator $L_{G}$ and the loss of discriminator $L_{D}$.

#### 3.3.1. Fuse-Generator Loss $L_{G}$

The Fuse-Generator loss includes content loss $L_{G}^{con}$ and adversarial loss $L_{G}^{adv}$, which are used to extract and reconstruct the basic intensity information accompanying the optimal texture details and restore the thermal infrared salient targets. With the weight λ controlling the trade-off between two terms, the Fuse-Generator is defined as follows:

(2) $$L_{G}=\lambda L_{G}^{con}+L_{G}^{adv}.$$

Among them, the content loss $L_{G}^{con}$ has two parts: basic-content loss $L_{SSIM}$ for extracting and reconstructing the basic intensity information, the max-gradient loss $L_{gra}$ for obtaining the optimal texture details, which is formulated as follows:

(3) $$L_{G}^{con}=L_{SSIM}+\eta L_{gra},$$

where the η is used to tradeoff the balance of intensity information and gradient variation.

Specifically, the $L_{SSIM}$ is formalized as follows:

(4) $$L_{SSIM}=\omega \left(1-SSIM_{VI,I_{F}}\right)+1-SSIM_{IR,I_{F}},$$

where the ω is employed to tradeoff the balance of intensity information and gradient variation. The $SSIM_{X,F}$ is the metric to measure the similarity between two images, including three different factors of brightness, contrast and structure, which is mathematically defined as follows:

(5) $$SSIM_{X,F}\;=\;\sum_{x,f}\;\frac{2\mu_{x}\mu_{f}\;+\;C_{1}}{\mu_{x}^{2}\;+\;\mu_{f}^{2}\;+\;C_{1}}\;\cdot \;\frac{2\sigma_{x}\sigma_{f}\;+\;C_{2}}{\sigma_{x}^{2}\;+\;\sigma_{f}^{2}\;+\;C_{2}}\;\cdot \;\frac{\sigma_{xf}\;+\;C_{3}}{\sigma_{x}\sigma_{f}\;+\;C_{3}},$$

where *X* and *F* in our work refer to the source image and fused image, respectively. The *x* and *f* mean the image patches of source image *X* and fused image *F*, μ and σ are the average values and the standard deviation. $C_{1}$, $C_{2}$ and $C_{3}$ are the parameters to make the metric stable.

Only the basic-content loss $L_{SSIM}$ will cause the issue of information attenuation in texture details. Therefore, we further employ the max-gradient loss $L_{gra}$ to obtain the optimal texture details. $L_{gra}$ is mathematically formalized as follows:

(6) $$L_{gra}=\frac{1}{HW}{\left(\nabla I_{f}-g_{\max}\right)}^{2},$$

where *H* and *W* are the height and width of the source images. ∇(·) refers to the step of calculating the gradient map. The idea of the loss $L_{gra}$ is to make the gradient map of the fused image ($\nabla I_{f}$) and the optimal gradient map of the source images $g_{\max}$ tend to be infinitely similar. The $g_{\max}$ is mathematically defined as follows:

(7) $$g_{\max}=round\left(\frac{\nabla I_{1}+\nabla I_{2}}{|\nabla I_{1}+\nabla I_{2}+eps|}\right)*\max(|\nabla I_{1}|,|\nabla I_{2}\left|\right),$$

where round(·) and max(·) mean the operations of rounding and taking the maximum value. The eps is a very small value to prevent the denominator from being 0.

The adversarial loss $L_{G}^{adv}$ is to further restore the thermal infrared salient targets from the source IR image in the fused image, which is defined as:

(8) $$L_{G}^{adv}=E\left[\log\left(1-D\left(I_{F}⊗M\right)\right)\right],$$

where *M* is the labeled mask reflecting the domains of salient thermal targets. When minimizing $L_{G}^{adv}$, $I_{F}⊗M$ is encouraged to be realistic enough and close enough to real data, i.e., $I_{R}⊗M$ to fool the discriminator *D*.

#### 3.3.2. Loss of Discriminator $L_{D}$

The discriminator loss $L_{D}$ is the term that forms an adversarial relationship with the Fuse-Generator adversarial loss $L_{G}^{adv}$. The $L_{D}$ is formulated as follows:

(9) $$L_{D}=E\left[-\log\left(D\left(IR⊗M\right)\right)\right]+E\left[-\log\left(1-D\left(I_{F}⊗M\right)\right)\right].$$

## 4. Experimental Results and Analysis

In this section, in order to show the superiority of our proposed FusionADA, we firstly compare it with 7 state-of-the-art fusion methods on the publicly available dataset RoadScene (https://github.com/hanna-xu/RoadScene (accessed on 1 December 2021)) qualitatively. Furthermore, we employ 8 metrics to evaluate their fusion results through qualitative comparisons. In addition, the ablation experiment of the adversarial learning is conducted. Finally, we show the fusion results of our FusionADA on another publicly available dataset TNO (https://figshare.com/articles/TNO_Image_Fusion_Dataset/1008029 (accessed on 1 December 2021)) dataset.

### 4.1. Experimental Settings

**Dataset and Training Details.** The training dataset is 45 aligned infrared and visible image pairs with different scenes selected from RoadScene. In order to improve the training performance, the tailoring and decomposition are applied as the expansion strategies before training to obtain a larger dataset. Specifically, the training dataset is uniformly cropped to 4736 patch pairs of size 128×128. There are 30 image pairs for testing. In the test phase, only the trained generator is used to generate the fusion image, and the input source images only need to be of the same resolution size.

Since this work is based on the adversarial learning of the generative adversarial network, we design a training strategy to keep the stability of the generative adversarial network in order to balance the adversarial relationship between the generator and the discriminator. The overall idea lies in that finding the loss value when the generator and the discriminator are in balance, and optimizing the generator or the discriminator to achieve their respective loss values through variable optimization times. The detailed training details of FusionADA are summarized in Algorithm 1. The λ, η, and ω are set to 3, 100 and 1.23 in Equations (2)–(4), respectively.
**Algorithm 1:** Training details of FusionADA Parameter definitions $N_{G}$, $N_{D}$: The numbers of steps for training *G*, *D*.
 $L_{max}$, $L_{min}$ and $L_{Gmax}$ are applied to determine a range when training.
 $L_{max}$ and $L_{min}$ mean the adversarial losses of *G* and *D*.
 $L_{Gmax}$: the total loss of *G*.
 We set $L_{max}=1.387$, $L_{min}=1.386$, and $L_{Gmax}=0.1$ in the first batch empirically in our work.1Initialize $\theta_{G}$ for *G*; $\theta_{D}$ for *D*.2For each training iteration:3**Train Discriminator***D*:
Sample *n* VI patches {$V^{1},\cdots ,V^{n}$} and *n* corresponding IR patches {$I^{1},\cdots ,I^{n}$};Acquire generated data {$F^{1},\cdots ,F^{n}$}Update Discriminator parameters $\theta_{D}$ by GradientDescentOptimizer to minimize $L_{D}$ in Equation (9); (**step I**)While $L_{D}>L_{max}\;\;\mathrm{and}\;\;N_{D}<10$, repeat **step I**. $N_{D}\leftarrow N_{D}+1$;**Train Generator***G*:Sample *n* VI patches {$V^{1},\cdots ,V^{n}$} and *n* corresponding IR patches {$I^{1},\cdots ,I^{n}$};Acquire generated data {$F^{1},\cdots ,F^{n}$}Update parameters $\theta_{G}$ by RMSPropOptimizer for minimizing $L_{G}$ in Equation (2); (**step II**)While $L_{D}<L_{min}\;\;\mathrm{and}\;\;N_{G}<10$, repeat **step II**. $N_{G}\leftarrow N_{G}+1$;While $L_{G}>L_{Gmax}\;\;\mathrm{and}\;\;N_{G}<10$, repeat **step II**. $N_{G}\leftarrow N_{G}+1$;


### 4.2. Comparison Algorithms and Evaluation Metrics

In order to verify the effectiveness of our FusionADA, we show some intuitive results from our work with 7 other state-of-the-art infrared and visible fusion methods, containing gradient transfer fusion (GTF) [24], fourth-order partial differential equations (FPDE) [25], hybrid multi-scale decomposition (HMSD) [26], DenseFuse [19], proportional maintenance of gradient and intensity (PMGI) [27], unified unsupervised image fusion (U2Fusion) [28], and generative adversarial network with multi-classification constraints (GANMcC) [29]. Among them, GTF, FPDE and HMSD are fusion methods based on the traditional framework, while DenseFuse, PMGI, U2Fusion and GANMcC are deep learning-based fusion methods. Besides the intuitive evaluation, to do a more accurate evaluation of the fused results, we employ eight metrics to evaluate the fusion performance of these eight fusion methods, including standard deviation (SD) [30], spatial frequency (SF) [30], entropy (EN) [31], mean gradient (MG) and edge intensity (EI) [32] that measure the fused image itself, feature mutual information (FMI), the sum of the correlations of differences (SCD) [33], and visual information fidelity (VIF) [33] that measure the correlation between the fused image and source images. Specifically, SD, SF, EN, MG and EI are used to evaluate the contrast, frequency, amount of information, details, gradient amplitude of the edge point in the fused image, respectively. FMI is used to evaluate the amount of feature information that is transferred from source images to the fused image. SCD and VIF are used to measure the sum of the correlations of differences and information fidelity, respectively.

### 4.3. Qualitative Comparisons

**Figure 2:**
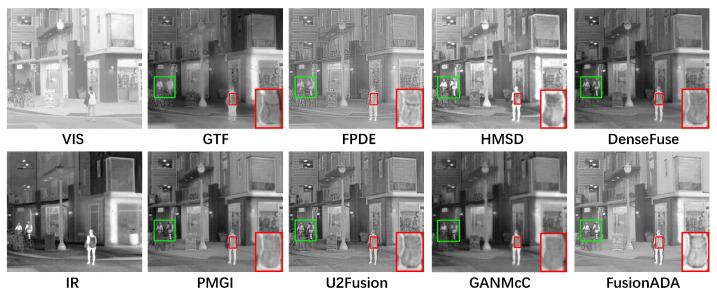
Qualitative comparison of FusionADA with corresponding seven state-of-the-art methods on the “building” image pair from the RoadScene dataset. From left to right, from top to bottom: source VIS image, the fused results of gradient transfer fusion (GTF), fourth-order partial differential equations (FPDE), hybrid multi-scale decomposition (HMSD) and DenseFuse. source IR image, the fused results of PMGI, U2Fusion, GANMcC and our FusionADA.

**Figure 3:**
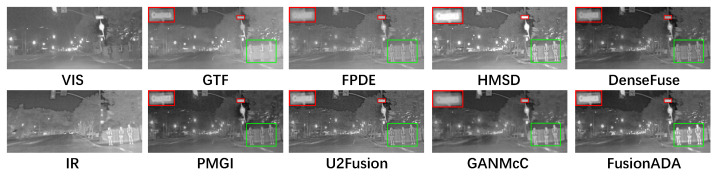
Qualitative comparison of FusionADA with corresponding seven state-of-the-art methods on the “crossroad 1” image pair from the RoadScene dataset.

**Figure 4:**
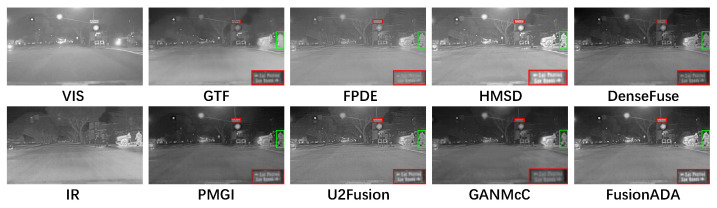
Qualitative comparison of FusionADA with corresponding seven state-of-the-art methods on the “crossroad 2” image pair from the RoadScene dataset.

**Figure 5:**
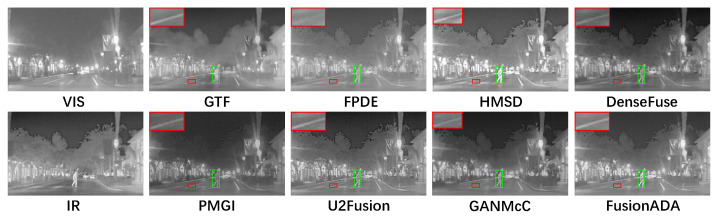
Qualitative comparison of FusionADA with corresponding seven state-of-the-art methods on the “road” image pair from the RoadScene dataset.

There are four representative and intuitive fusion results of eight methods on infrared and visible images from the RoadScene dataset in Figure 2, Figure 3, Figure 4 and Figure 5. Compared to the existing seven other comparative fusion methods, our fused results show three obvious advantages. First, The salient thermal targets can be characterized clearly in our fused images, such as the pedestrians in Figure 2, Figure 3 and Figure 5, and the driver and passenger who got off the bus halfway in Figure 4 (all shown in the green boxes). The targets in the fused results of other methods all look dimmer compared to our results. Due to the salient thermal targets in our fused images, the drivers and machines can identify targets more easily and accurately, which facilitates the subsequent operations. Second, the scenes in our fused results show richer texture details, such as the schoolbag in Figure 2, the signs in Figure 3 and Figure 4 and the pavement marking in Figure 5 (all shown in the enlarged red boxes). Some scenes in the results of other methods seem fuzzier. The rich texture details are more conducive to scene understanding for the drivers and machines. Last but not the least, our results look cleaner than others without redundant fog or noise compared with the results of other methods.

### 4.4. Quantitative Comparisons

To have a more comprehensive and objective evaluation of the experimental results. We selected 30 test pairs of infrared and visible images randomly to further perform quantitative comparisons of our FusionADA with the competitors on eight fusion metrics. Each test image pair is aligned with the same resolution. The results of their values are summarized in Table 2. It is worth noting that our FusionADA can almost reach the optimal or suboptimal mean values on the eight metrics. For the metrics MG, EI, FMI and SCD, our FusionADA can also achieve comparable results with the suboptimal average values, only following behind a certain method by a narrow margin. It can be concluded that our results contain stronger contrast, richer texture details, more information and are closer to source images with less distortion.

In addition, we also provide the mean and standard deviation of runtime for eight methods in Table 3. Although our FusionADA does not achieve optimal efficiency, it still plays a comparable role.

### 4.5. Ablation Experiment of Adversarial Learning

**Figure 6:**
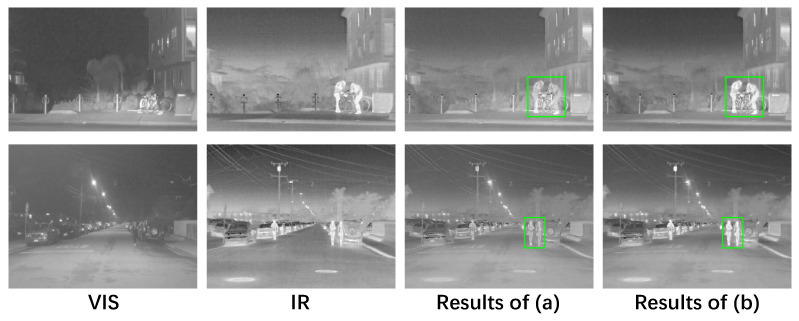
Results on whether the adversarial learning exists. From left to right: source VIS image, source IR image, the fused results without adversarial learning, the fused results of our FusionADA (with adversarial learning).

The adversarial learning with a labeled mask is further employed in our FusionADA to restore the salient thermal targets from the source infrared image. To show the effect of adversarial learning, the following comparative experiments are conducted: (a) the adversarial learning is not applied; (b) the adversarial learning is applied. The experimental settings in other parts of the ablation experiments are the same. As can be seen from Figure 6, the fused results with adversarial learning own more salient thermal targets, which is more beneficial to the drivers and machines to identify the targets. Therefore, we can conclude that adversarial learning plays an important part in the fusion process.

### 4.6. Generalization Performance

**Figure 7:**
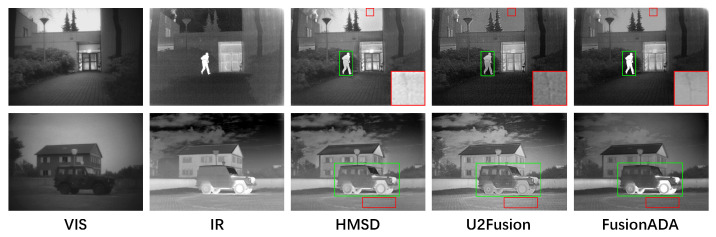
Fusion results on the TNO dataset.

Our FusionADA also performs well on other datasets. To show the performance of our FusionADA on other datasets, we choose the TNO dataset to carry out the experiments without retraining the fusion methods. In particular, we choose two state-of-the-art methods HMSD and GANMcC that perform well on RoadScene to be the comparative methods with our FusionADA. The intuitive fusion results are presented in Figure 7. By contrast, with less noise, the salient thermal targets can be characterized more clearly and the richer texture details are contained in our results, which can be concluded that our FusionADA has good generalization performance and obtains excellent fusion results on other datasets.

## 5. Conclusions

In this paper, we propose a novel end-to-end fusion model for advanced driving assistance to obtain key and rich visual information under sophisticated road conditions, called FusionADA. Specifically, we achieve our FusionADA by fusing the infrared and visible images from infrared and visible sensors. For drivers and machines, based on the scenes, the salient thermal targets and rich texture details are indispensable for identifying targets easily and accurately. Therefore, in our model, we guide the generator to generate the optimal texture details from source images. Meanwhile, we constitute an adversarial framework with a labeled mask to further restore the salient thermal targets from the source infrared image. In addition, our FusionADA is achieved in a fully end-to-end way, which avoids manually designing complicated activity level measurements and fusion rules. The adequate experimental results reveal that our FusionADA not only presents better visual performance compared with other state-of-the-art methods, but also preserves the maximum or approximate maximum amount of features from source images.

## Figures and Tables

**Table 1:** Input/output channels of all convolutional layers.

		Number of Input Channels	Number of Output Channels
Encoder	Convolutional layer 1 of branch 1/2	1	16
	Convolutional layer 2 of branch 1/2	16	16
	Convolutional layer 3 of branch 1/2	32	16
	Convolutional layer 4 of branch 1/2	48	16
	concatenation	128	
Decoder	Convolutional layer 1	128	64
	Convolutional layer 2	64	32
	Convolutional layer 3	32	1

**Table 2:** Quantitative comparison of our FusionADA for infrared and visible image fusion with 5 other comparative methods. The average and standard deviation values of eight metrics for different methods are provided; red: optimal average values, blue: suboptimal average values.

	SD	SF	EN	MG	EI	FMI	SCD	VIF
GTF	0.1823±0.0712	0.0295±0.0117	7.3142±0.5122	0.0118±0.0088	0.1083±0.0321	0.8863±0.0452	0.9835±0.0425	0.5313±0.1532
FPDE	0.1337±0.0712	0.0393±0.0821	6.9404±0.5213	0.0200±0.0400	0.1668±0.3251	0.8659±0.0635	1.0919±0.6356	0.4991±0.2985
HMSD	0.1741±0.1029	0.0497±0.0212	7.3015±2.3251	0.0210±0.1254	0.2423±0.1254	0.8701±0.2563	1.4882±0.5632	0.8572±0.2153
DenseFuse	0.1733±0.1325	0.0398±0.0215	7.2950±0.3261	0.0192±0.0071	0.1625±0.0512	0.8749±0.0421	1.6316±0.2123	0.7597±0.7235
PMGI	0.1519±0.0852	0.0399±0.0212	7.1091±0.4213	0.0188±0.0057	0.1655±0.0564	0.8718±0.0432	1.2878±0.8421	0.7424±0.5231
U2Fusion	0.1625±0.0721	0.0493±0.0164	7.2328±0.5231	0.0237±0.0085	0.2116±0.1021	0.8716±0.0421	1.4837±0.4351	0.9337±0.9013
GANMcC	0.1702±0.0632	0.0341±0.0123	7.2345±1.2362	0.0163±0.0632	0.1506±0.0965	0.8605±0.3526	1.5819±0.3215	0.6832±0.1321
FusionADA	0.1863±0.0753	0.0509±0.0301	7.3456±0.2365	0.0221±0.0102	0.1851±0.1212	0.8801±0.0462	1.5825±0.3251	0.9619±0.6324

**Table 3:** The average and standard deviation of running time for eight methods. (unit: second).

	GTF	FPDE	HMSD	DenseFuse	PMGI	U2Fusion	GANMcC	FusionADA
Running Time	2.72±1.20	0.95±0.38	0.59±0.15	0.29±0.04	0.14±0.03	0.98±0.45	0.295±0.32	0.10±0.01

## Data Availability

Not applicable.
